# Wideband tympanometry as a noninvasive marker of intracranial pressure changes: a postural study in healthy adults

**DOI:** 10.3389/fneur.2026.1874870

**Published:** 2026-07-30

**Authors:** Florian Nourrisson, Tuan Le Van, Franck Marzani, Muhammad Umer, Leixin Nie, Alexis Bozorg Grayeli

**Affiliations:** 1 Department of Otolaryngology—Head and Neck Surgery, Dijon University Hospital, Dijon, France; 2 Department of Neurosurgery, Dijon University Hospital, Dijon, France; 3 ImViA UR 7535, Université Bourgogne Europe, Dijon, France; 4 ICMUB, UMR CNRS 6302, Université Bourgogne Europe, Dijon, France

**Keywords:** biomarker, inner ear mechanics, intracranial pressure, neuro-otology, noninvasive monitoring, posture, wideband tympanometry

## Abstract

**Background:**

Reliable noninvasive assessment of intracranial pressure (ICP) remains an unmet clinical need. Wideband tympanometry (WBT), which reflects middle ear mechanics, may provide indirect information on ICP-related inner ear pressure changes.

**Objective:**

To determine whether postural ICP variations induce measurable changes in WBT parameters in healthy adults.

**Methods:**

In this prospective study, 21 healthy adults (42 ears) underwent repeated WBT measurements in standing, supine, and Trendelenburg (−17°) positions. Five measurements were performed per ear in each position. Parameters analyzed included peak pressure (PP), peak pressure difference (*ΔP*), resonant frequency (RF), duplication frequency (DF), and absorbance-derived metrics. Statistical comparisons were performed using paired non-parametric tests with Šidák correction for multiple comparisons.

**Results:**

A total of 626 valid recordings were analyzed. PP and *ΔP* significantly increased in the Trendelenburg position compared with standing (PP: −10.9 ± 8.1 vs. 5.5 ± 24.4 daPa, *p* < 0.01; *ΔP*: 90.7 ± 42.0 vs. 105.0 ± 51.5 daPa, *p* < 0.05). No significant changes were observed for RF, DF, or absorbance-derived parameters. Interaural reproducibility was moderate to high for most variables but low for *ΔP*.

**Conclusion:**

Postural changes associated with presumed ICP elevation induce measurable modifications in WBT signals, particularly PP and *ΔP*. These findings support WBT as a promising noninvasive approach for detecting ICP variations. Further validation against invasive ICP monitoring is warranted.

## 1 Introduction

Intracranial pressure (ICP) is a major physiological determinant of cerebral perfusion and neurological outcome in critically ill patients (1–3). Intracranial hypertension contributes to secondary brain injury and remains a central target of neurocritical care management (4, 5).

Although invasive ICP monitoring remains the clinical reference standard, it requires neurosurgical access and carries risks including hemorrhage and infection (6, 7). Consequently, the development of reliable noninvasive approaches for ICP assessment remains an important unmet clinical need.

Several noninvasive techniques have been proposed, including neuroimaging, transcranial Doppler ultrasonography, fundoscopy, electrophysiological monitoring, and otoacoustic emission analysis (1, 8–11). However, none has yet achieved sufficient reliability for routine replacement of invasive monitoring.

Body posture is known to influence ICP. ICP increases from the standing position to supine posture and further increases during head-down tilt (12–14). Previous studies demonstrated that posture-related ICP changes may influence cochlear and middle ear micromechanics (8–10, 13, 15). The physiological basis for this relationship likely involves pressure transmission between the cerebrospinal fluid compartment and the inner ear through the cochlear aqueduct and vestibular aqueduct (16–19). These mechanisms may alter stapes mobility, annular ligament tension, and cochlear fluid dynamics.

Wideband tympanometry (WBT) is an emerging technique capable of characterizing sound energy transfer across a broad frequency spectrum (20, 21). Because WBT is sensitive to middle and inner ear mechanical properties, it may provide indirect information regarding ICP-related pressure changes. WBT abnormalities have already been described in Ménière’s disease and endolymphatic hydrops, conditions associated with altered inner ear pressure regulation (22–25). More recently, ICP-related changes in WBT signals have been suggested in experimental postural studies (26, 27). In this context, WBT could represent a rapid, bedside, noninvasive biomarker for ICP-related physiological changes.

The primary objective of this study was therefore to identify WBT parameters modified by posture-induced ICP variations in healthy adults. The secondary objective was to evaluate interaural reproducibility of these measurements.

## 2 Materials and methods

### 2.1 Study design and participants

This prospective study included 21 healthy adult volunteers without relevant medical history. The study protocol was approved by the institutional ethics committee (CCP EST III, No. 2021-A00035-36). Written informed consent was obtained from all participants. Participants underwent otoscopy, pure-tone audiometry, and speech audiometry before inclusion.

The inclusion criteria were:

- Age >18 years
- Volunteer and capable of fully understanding and cooperating during the protocol
- No past medical history of otolaryngological, neurological or cardiovascular diseases
- No reported hearing loss

The exclusion criteria were:

- Hearing loss at inclusion audiometry (pure-tone average > 20 dB, and/or SDS < 90%)
- Abnormal otoscopic findings

### 2.2 Experimental protocol

**Figure 1:**
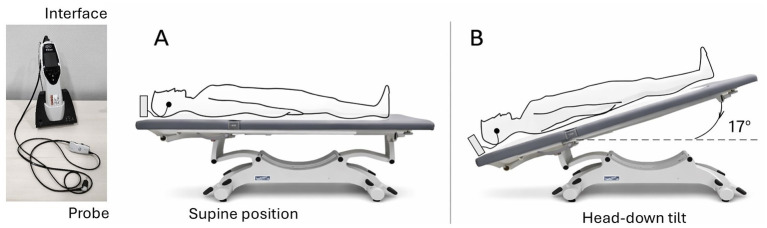
Experimental setup and body positioning. **(A)** Supine position on the examination table with the tympanometry probe inserted in the external auditory canal. **(B)** Head-down tilt position at 17°, illustrating the change in body inclination relative to the horizontal plane.

WBT measurements were performed in three body positions (Figure 1):

- Standing
- Supine
- Trendelenburg position (−17°)

For each position, five repeated measurements were obtained in both ears using an electrically adjustable examination table (Quest Promotal®, Ernée, France). The standing position was always tested first, followed by supine and Trendelenburg positions. The first measurement was performed 5 min after achieving the target position. The probe was removed and reinserted before each recording. In case of signal abnormality (incomplete sweep, pressure leak, noise, …), the recording was discarded and immediately repeated.

### 2.3 Wideband tympanometry measurements

Measurements were obtained using the Titan Suite™ device equipped with the IMP 440 module (Interacoustics®, Middlefart, Denmark). The external auditory canal was pressurized from +200 to −300 daPa while frequencies ranging from 226 to 8,000 Hz were delivered (28, 29).

**Figure 2:**
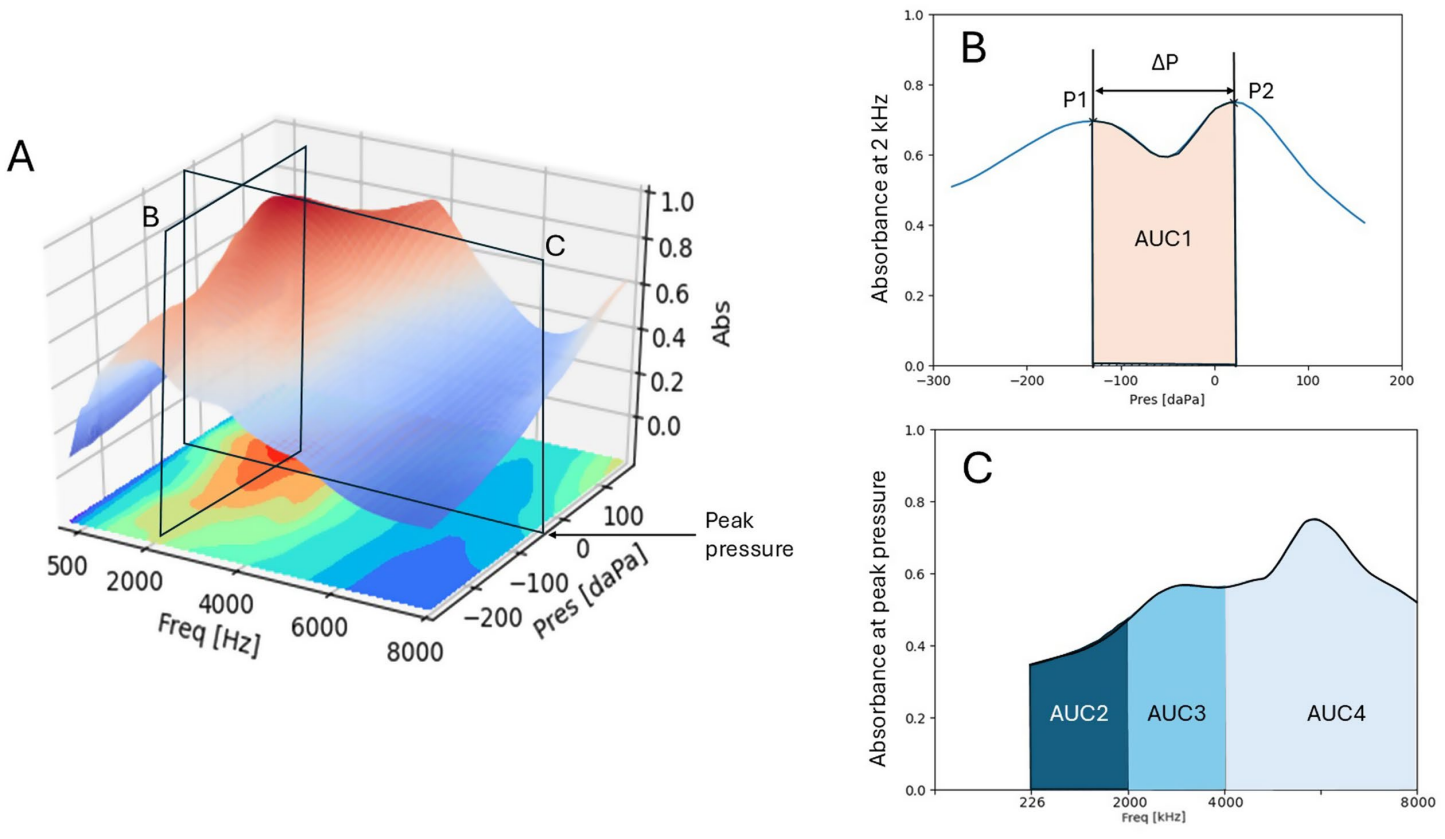
Extraction of peak-to-peak pressure distance from wideband tympanometry. **(A)** Three-dimensional representation of absorbance as a function of ear canal pressure and frequency. **(B)** Absorbance as a function of pressure at 2 kHz showing the two local maxima used to calculate *ΔP*. **(C)** Areas under the absorbance curve as a function of the frequency range: AUC2-4.

The following parameters were analyzed:

- Equivalent ear volume (EEV, ml) corresponding to the estimated external ear-canal volume
- Peak pressure difference (*ΔP*, daPa, Figure 2) representing the difference of pressure between the absorbance peaks at 2 kHz
- Area under the absorbance curve (as a function of pressure ranging from −280 to +160 daPa) between the 2 peaks at 2 kHz (AUC1, in arbitrary units)
- Maximum absorbance curve frequency (MACF, Hz) representing the frequency at which the absorbance curve as a function of pressure yields the maximum AUC in the range of 800–5,000 Hz.
- Duplication frequency (DF, Hz): the frequency at which the absorbance curve as a function of pressure shows 2 distinct peaks
- Peak pressure (PP, daPa): represents the pressure at which the area under the absorbance curve is the largest, in the range of −280 to +160daPa
- Resonant frequency (RF, Hz): the lowest available resonant frequency at PP
- AUC2 (aera under the absorbance curve at PP as a function of frequency < 2 kHz), AUC3 (area under the same curve in the range of 2–4 kHz), AUC4 (area under the curve > 4 kHz, Figure 2C)
- Relative AUC2–4 represent the proportions of AUC2, AUC3 and AUC4 to total AUC

### 2.4 Statistical analysis

Sample size calculation was performed using G*Power (30). Based on previous tympanometric studies (31, 32), a sample size of 21 participants was estimated to provide 95% power with *α* = 0.05.

Quantitative variables were expressed as mean ± standard deviation. Comparisons were performed using the Wilcoxon signed-rank test with Šidák correction for multiple comparisons. Interaural agreement was evaluated using Pearson’s correlation coefficient.

Statistical significance was defined as *p* < 0.05.

## 3 Results

### 3.1 Population characteristics

Twenty-one healthy subjects (42 ears) were included. Mean age was 33.6 ± 13.8 years. No participant presented hearing loss or tympanic membrane abnormality.

A total of 682 WBT recordings were attempted. Fifty-two recordings (7%) were excluded during acquisition because of artifacts or technical errors, 4 (1%) were excluded secondarily due to inconsistency or incomplete frequency or pressure sweep, resulting in 626 valid recordings for analysis. At 2 kHz, absorbance curves showed two identifiable peaks in 94% of recordings.

### 3.2 Right–left comparison

No significant difference was observed between right and left ears for any analyzed parameter (33). Moderate-to-high interaural correlations were observed for most variables, particularly EEV, PP, MACF, AUC2–4, and relative AUC2–4. In contrast, *ΔP* showed poor interaural correlation (Table 1).

**Table 1:** Interaural comparison of wideband tympanometry parameters averaged across body positions.

Parameter	Right ear	Left ear	Pearson’s *r*
EEV (mL)	1.40 ± 0.26 (0.8–2.2)	1.40 ± 0.26 (0.8–1.9)	0.78
RF (Hz)	647.4 ± 131.8 (355.4–889.3)	650.1 ± 117.9 (420.8–1072.0)	0.36
PP (daPa)	−3.9 ± 36.1 (−162 to 61.5)	−1.7 ± 36.1 (−160.8 to 127.2)	0.76
*ΔP* (daPa)	98.7 ± 51.2 (30–260)	84.4 ± 40.8 (20–190)	0.22
AUC1	60.9 ± 35.1 (12.6–183.1)	52.1 ± 28.7 (5.7–125.6)	0.43
AUC2	339,616 ± 69,367 (147,552–485,259)	342,681 ± 82,754 (139,843–530,826)	0.84
Relative AUC2	0.20 ± 0.06 (0.08–0.50)	0.20 ± 0.08 (0.09–0.53)	0.67
AUC3	524,879 ± 112,556 (216,686–706,865)	503,628 ± 119,789 (195,054–718,051)	0.65
Relative AUC3	0.30 ± 0.06 (0.18–0.46)	0.30 ± 0.06 (0.20–0.40)	0.82
AUC4	699,006 ± 240,613 (195,480–1,254,276)	642,471 ± 242,395 (143,924–1,232,832)	0.76
Relative AUC4	0.40 ± 0.09 (0.20–0.60)	0.40 ± 0.11 (0.10–0.60)	0.80
MACF (Hz)	1,349 ± 867 (817–4,896)	1,389 ± 894 (817–4,896)	0.95
DF (Hz)	1,863 ± 714 (917–3,462)	1,877 ± 767 (866–4,362)	0.44

Values are presented as mean ± SD (range). No significant interaural difference was observed for any parameter (Wilcoxon signed-rank test, all *p* > 0.05).

AUC, area under the curve; DF, duplication frequency; EEV, equivalent ear volume; MACF, maximum absorbance curve frequency; PP, peak pressure; RF, resonant frequency; *ΔP*, pressure difference between the two absorbance peaks at 2 kHz.

### 3.3 Effect of body position

Postural changes did not significantly affect EEV, RF, MACF, DF, or absorbance-derived parameters (Table 2).

**Table 2:** Effect of body position on wideband tympanometry parameters.

Parameter	Standing	Supine	Trendelenburg
EEV (mL)	1.42 ± 0.27	1.41 ± 0.26	1.45 ± 0.23
RF (Hz)	679.5 ± 122.3	641.4 ± 91.1	625.4 ± 90.3
PP (daPa)	−10.9 ± 8.1	−3.1 ± 36.9	5.5 ± 24.4**
*ΔP* (daPa)	90.7 ± 42.0	88.5 ± 32.7	105.0 ± 51.5*
AUC1	54.0 ± 27.3	54.4 ± 25.4	66.5 ± 38.6
AUC2	338.4 ± 64.3	337.6 ± 76.2	347.4 ± 80.5
Relative AUC2	0.24 ± 0.07	0.23 ± 0.07	0.23 ± 0.06
AUC3	506.5 ± 111.5	501.8 ± 107.4	534.5 ± 99.9
Relative AUC3	0.30 ± 0.10	0.30 ± 0.10	0.30 ± 0.10
AUC4	648.6 ± 220.6	672.9 ± 243.0	679.0 ± 238.3
Relative AUC4	0.40 ± 0.10	0.40 ± 0.10	0.40 ± 0.10
MACF (Hz)	1384.4 ± 865.5	1406.3 ± 928.1	1317.1 ± 856.4
DF (Hz)	1939.2 ± 632.8	1830.1 ± 663.7	1839.9 ± 614.0

Values are presented as mean ± SD (*n* = 21). Statistical comparisons were performed using the Wilcoxon signed-rank test with Šidák correction for multiple comparisons.

*p < 0.05 vs. standing position; **p < 0.01 vs. standing position.

EEV, equivalent ear volume; RF, resonant frequency; PP, peak pressure; *ΔP*, pressure difference between the two absorbance peaks at 2 kHz; AUC1, Area under the absorbance curve (as a function of pressure ranging from −280 to +160 daPa) between the 2 peaks at 2 kHz (AUC1, in arbitrary units); AUC2 (aera under the absorbance curve at PP as a function of frequency <2 kHz); AUC3 (AUC in the range of 2–4 kHz); AUC4 (AUC > 4 kHz); MACF, Maximum absorbance curve frequency (Hz); DF, Duplication frequency (DF, Hz), Relative AUC2, 3, and4 (AUC2, 3 or 4/total AUC at PP).

By contrast, both PP and *ΔP* significantly increased in the Trendelenburg position compared with the standing position:

- PP: −10.9 ± 8.1 vs. 5.5 ± 24.4 daPa (*p* < 0.01)
- *ΔP*: 90.7 ± 42.0 vs. 105.0 ± 51.5 daPa (*p* < 0.05)

No significant differences were observed between standing and supine positions.

## 4 Discussion

This study demonstrates that posture-related ICP changes are associated with measurable modifications in WBT signals in healthy adults.

Among all investigated parameters, PP and *ΔP* were the most sensitive to posture-induced ICP variations. These findings support previous observations suggesting that ICP influences middle and inner ear biomechanics.

The biological plausibility of this relationship is supported by anatomical communication pathways between the cerebrospinal fluid compartment and the inner ear through the cochlear aqueduct and vestibular aqueduct (16–19). Increased ICP may alter intracochlear pressure, annular ligament tension, and stapes mechanics, thereby modifying sound transmission properties measurable by WBT.

Our results are consistent with previous work by Franco-Vidal et al., who reported widening of absorbance peak separation during head-down tilt (26). Similarly, Torrecilla et al. (27) suggested that WBT modifications associated with ICP elevation may resemble those observed in Ménière’s disease (34).

Interestingly, PP demonstrated significant posture-related variation despite relatively high inter-individual variability. This suggests that PP may represent a sensitive marker of ICP-related mechanical changes. Likewise, the increase in *ΔP* observed in the Trendelenburg position supports the hypothesis that posture-induced ICP elevation modifies the mechanical behavior of the middle and inner ear system. Although PP and *ΔP* are both derived from the wideband absorbance curve, they reflect fundamentally different biomechanical properties of the middle ear and should not be expected to behave similarly in terms of interaural symmetry. PP represents the pressure at which maximal acoustic energy absorption occurs across the tested frequency range. It reflects the overall optimal static pressure condition for the middle ear mechanical elements—primarily the tympanic membrane and ossicular chain—to efficiently transmit acoustic energy (35). This parameter is relatively robust, as it is derived from the broadband absorbance curve as a whole, and its moderate-to-high interaural correlation is consistent with the known bilateral symmetry of middle ear static mechanics in healthy individuals. In contrast, *ΔP* represents the pressure difference between the two absorbance peaks observed at 2 kHz. This parameter is thought to reflect the mechanical properties of the stapedial annular ligament—particularly its stiffness—which governs the fine pressure-dependent tuning of the absorbance notch at this frequency. Critically, the double-peak pattern at 2 kHz is not a consistent feature across all subjects; it is absent or poorly defined in a non-negligible proportion of individuals. When this morphological feature is inconstant, any derived measurement will inherently carry greater variability, and interaural correlation will be reduced regardless of true physiological symmetry. Therefore, the apparent contradiction between the moderate-to-high interaural correlation of PP and the poor interaural correlation of *ΔP* is not paradoxical but rather reflects the differing robustness and anatomical substrates of these two parameters. This distinction also highlights an important limitation: relying solely on *ΔP* as a surrogate marker for intracranial pressure estimation may be unreliable due to its inherent inter-individual and intra-individual variability.

By contrast, RF, DF, and absorbance-derived parameters did not significantly change with posture. Several explanations are possible. First, the ICP increase induced by the postural protocol may have remained modest in healthy subjects. Second, the 5-min equilibration period may have been insufficient in some individuals because pressure transmission through the cochlear and vestibular aqueducts may occur with variable delay. Third, repeated probe repositioning may have increased measurement variability.

From a clinical perspective, these findings are particularly relevant because WBT is rapid, noninvasive, inexpensive, and widely available in otological practice (36–38). If validated against invasive ICP monitoring, WBT could potentially contribute to bedside ICP screening or longitudinal monitoring in selected neurological settings.

Potential applications may include:

- Neurocritical care monitoring,
- Idiopathic intracranial hypertension,
- Shunt monitoring,
- Aerospace medicine,
- And perioperative neurosurgical assessment.

The ability to detect ICP-related physiological changes through a simple otological examination could open new perspectives for noninvasive neuro-monitoring.

This study nevertheless has several limitations. First, ICP was not directly measured and posture was used only as an indirect surrogate model of ICP variation. Second, the sample size remained limited. Third, body position order was not randomized, potentially introducing sequence effects. Fourth, probe reinsertion may have contributed to measurement variability. Fifth, all measurements were obtained within a single recording session per participant, and the reproducibility analysis reported here addressed interaural (left–right) agreement rather than longer-term repeatability; day-to-day reproducibility of WBT parameters, including the posture-related changes in PP and *ΔP*, was not assessed and remains to be established. Although the immediate within-session test–retest reliability of wideband tympanometry has been reported to be excellent (39), the repeatability of multifrequency tympanometric components across separate sessions appears more variable (40), particularly for resonance-related measures (41); establishing the across-day stability of the parameters identified here is therefore an important prerequisite for any future longitudinal application.

Future studies should therefore compare WBT findings directly with invasive ICP monitoring in patients presenting pathological ICP variations, and should also formally establish the day-to-day test–retest reliability of WBT parameters, a prerequisite for their use in longitudinal or serial ICP monitoring. Larger datasets may additionally enable machine-learning approaches capable of integrating the multidimensional structure of WBT signals.

## 5 Conclusion

Wideband tympanometry signals appear to be influenced by posture-related ICP changes.

In healthy adults, the Trendelenburg position was associated with significant increases in PP and *ΔP*, supporting the hypothesis that WBT may provide indirect information regarding ICP-related inner ear mechanical changes.

These findings support the potential role of WBT as a noninvasive tool for ICP assessment. Further studies combining WBT with invasive ICP monitoring are needed.

## Data Availability

The raw data supporting the conclusions of this article will be made available by the authors, without undue reservation.
